# SELTP‐assembled battery drives totipotency of somatic plant cell

**DOI:** 10.1111/pbi.13107

**Published:** 2019-05-04

**Authors:** Huihui Guo, Haixia Guo, Li Zhang, Yijie Fan, Yupeng Fan, Fanchang Zeng

**Affiliations:** ^1^ State Key Laboratory of Crop Biology College of Agronomy Shandong Agricultural University Tai'an China

**Keywords:** cotton biotechnology, cell totipotency, somatic embryogenesis, SELTP, amyloplasts, cell polarity establishment, embryogenic fate determination

Plants display a remarkable capacity for somatic cell totipotency, as demonstrated by single plant cells that can develop into embryos and complete plants. How does a single somatic cell become a whole plant? This represents the forefront of the most compelling puzzles since Haberlandt's prediction of totipotency (1902) and is one of the top 25 big questions that face scientific inquiry today (Vogel, 2005). Elucidation of somatic cell totipotency is of great fundamental importance. Furthermore, studies in this line are fundamental for plant breeding and the improvement of plant productivity.

As a notable illustration of totipotency, somatic embryogenesis (SE) is quite interesting and provides an ideal system for investigation of the whole process of single cell differentiation as well as the expression of totipotency (Yang and Zhang, 2010; Zeng *et al*., 2007; Zimmerman, 1993). However, the underlying major cellular processes driving totipotency during SE are poorly understood. It is difficult to identify the cells capable of embryogenesis. Although an earlier study assumed that all plant cells were equally labile, only a subset of cells can transform into an embryogenic state. Investigations on the cellular features of the totipotency process are of great fundamental and practical importance in crop biotechnology.

During the somatic‐to‐embryogenic transition, cells must dedifferentiate, activate their cell division cycle and reorganize their metabolic and physiological states. This then results in the totipotency fate determination of somatic plant cells and embryogenesis initiation. The transition phase towards competent and embryogenic cell types is much less defined so far. Therefore, it is critical to track and dissect the specific cellular events associated with the acquisition of embryogenic competence in such highly refined systems.

Synchronous and high‐frequency SE systems, such as suspension cultures, are required for investigating the essential cellular basis involved in somatic‐to‐embryogenic transition in plants. Thus, we established such SE systems in this study. Large populations of homogenized plant cells can be easily obtained and handled in liquid culture. The flexibility and efficiency of the cell lines from the liquid culture system provided a useful tool for examining the fundamental process of totipotency.

Previous results indicated an SE‐associated lipid transfer protein (named SELTP) that was markedly activated in proembryogenic masses (PEMs; Zeng *et al*., 2006), which implied the critical role of the *LTP* gene in the initiation of SE. LTPs are basic and abundant transporter proteins in higher plants that contain a signal peptide and are usually secreted in the cell wall (Kader, 1996 *;* Sterk *et al*., 1991).In our current study, however, a subcellular localization assay revealed that the SELTP‐GFP fusion protein was specifically localized on the amyloplast membrane (Figure 1a–f). Starch grains were organized in amyloplasts that were assembled by SELTP. This finding differs from previous reports of the general localization pattern of LTPs in the cell wall that have a regular role in controlling cell wall expansion for the establishment of cell polarity.

**Figure 1:**
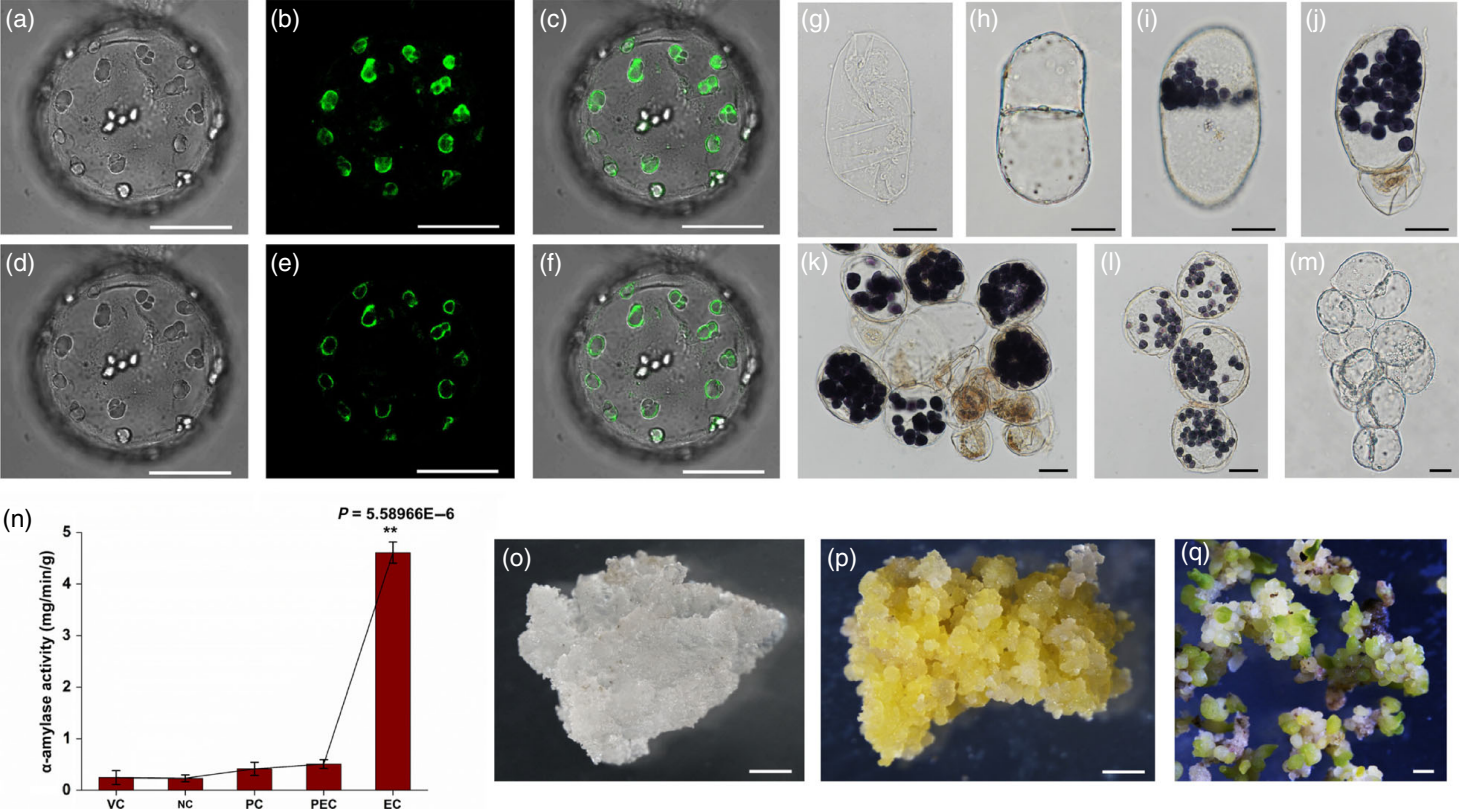
Cellular organization and activity of SELTP‐assembled amyloplasts during somatic‐to‐embryogenic transition. (a–f) SELTP specifically localized in the membrane of amyloplasts. (a) and (d) Brightfield; (b) and (e) green fluorescence of the SELTP‐GFP fusion protein; (c) and (f) merged. (a–c) represent images of multilayers by *z*‐axis scanning, and (d–f) represent mono‐layer images. Scale bars = 25 μm. (g–m) Cytological amyloplast activity patterns during cell transdifferentiation. (g) Vacuolated dedifferentiated cells in the early culture stage; (h) tubular nonembryogenic cells undergoing regular symmetrical division without amyloplasts; (i–m) cells under somatic‐to‐embryogenic transition. Cells with amyloplast polarity undergo asymmetrical division to form two different cells (i) and result in degeneration of the daughter cell without amyloplasts in (j). In the suspension cell population, cells devoid of amyloplasts resulting from asymmetrical division died, while cells full of amyloplasts possessed embryogenic potential in (k). Through subculture, the purified proembryogenic cells with amyloplasts can be obtained in (l), originating from the suspension cell population (k). Final transition to embryogenic cells with totipotency in (m), resulting from a sharp hydrolyzation of amyloplasts. Scale bars = 25 μm. (n) Cellular alpha‐amylase levels at typical transdifferentiation stages. VC, vacuolated cells in the early dedifferentiation stage; NC, nonembryogenic cells divided symmetrically without amyloplasts; PC, polarized cells with amyloplasts; PEC, proembryogenic cells full of amyloplasts; EC, embryogenic cells with totipotency resulting from a sharp hydrolyzation of amyloplasts. Notably, a dramatic increase in amylase activity was detected in EC. Each value is the mean ± standard error from three biologically independent measurements. Black asterisks indicate statistically significant differences between PEC and EC (*P* < 0.01). (o–q) Morphology of cultures proliferated from cells with different embryogenic fates. (o) Nonembryogenic calli; (p) embryogenic calli; (q) somatic embryos. Bars = 1 mm.

Using these systems, cellular organization and activity during transdifferentiation were analysed in living cells; these elements are crucial for proper cell functioning. Our results demonstrated that the somatic‐to‐embryogenic transition was divided into two different steps: the embryogenic pathway induction phase, followed by the phase of acquisition of embryogenic competence. Two new notable cellular events determining somatic‐to‐embryogenic transition during totipotency were identified in this study.

In the first induction phase, somatic dedifferentiated cells (Figure 1g) are divided predominantly by regular symmetrical division without amyloplasts (Figure 1h); in this case, only unorganized proliferation will occur, leading to nonembryogenic callus. A fraction of cells divided asymmetrically to form two different cells with SELTP‐assembled amyloplast polarities (Figure 1i). This resulted in degeneration of the cells without amyloplasts and tended to confer embryogenic competence to the cell with amyloplasts (Figure 1j). In the suspension cell population, the proembryogenic cells resulting from polarization were small and full of amyloplasts (Figure 1k). The purified cells with SELTP‐assembled amyloplasts were further obtained through cell tracking during subculture (Figure 1l).The cells went through an asymmetric cell division that led to daughter cells with different characteristics that were dependent on their further fate. The daughter cells that were devoid of amyloplasts died, while the cells with amyloplasts were embryogenic and further formed somatic embryos (Figure 1k, p, q). Our results revealed that somatic plant cells initiated an embryogenic pathway through an unequal first division parallel with the polarized amyloplasts assembled by SELTP.

Induction of embryogenic totipotency requires cell fate change through initiating division and establishing a new polarity in somatic cells. The typical cell polarization and the high amyloplast accumulation in our study were interpreted as the first observable steps towards the acquisition of a competent state. Concomitant with the somatic‐to‐embryogenic transition, the increase in the endogenous content of starch appeared to be related to an increasing demand for energy, which is an essential point in the conversion of embryogenic fate acquisition. Our results suggested that cellular polarities were driven by SELTP‐assembled amyloplasts in a single embryogenic cell precursor, which primed embryogenic pathway initiation.

In the second phase, our results revealed that as proembryogenic cells progressed, SELTP‐assembled amyloplasts crumbled away (Figure 1m) and degraded with a corresponding sharp increase in alpha‐amylase (Figure 1n). The degradation of the amyloplasts paralleled the increased activity of the amylase, which was a prominent feature during embryogenic cell fate determination. A transient increase in the amylase level and a sharp increase in starch energy released from SELTP‐associated amyloplasts, as a critical event, were associated with the activation of plant cell totipotency and the acquisition of embryogenic competence. The discharge of the SELTP‐associated battery caused by amylase surge, as a striking cellular physiological and metabolic process, was suggested as the essential factor for embryogenic cell fate determination.

Embryogenic fate was determined following the two cellular events of polarization and sharp energy release. The cells continued to proliferate and progress, forming perfect embryogenesis and embryoids in solid media culture (Figure 1p, q). However, dedifferentiated cells without amyloplasts under symmetrical division only processed unorganized proliferation, leading to nonembryogenic calli (Figure 1o).

In this study, successive amyloplast polarization, accumulation and breakdown in single cells were interpreted as the early reflection of somatic‐to‐embryogenic transition and acquisition of cell totipotency during SE. Cellular amyloplast polarity establishment was suggested as the primary driver for inducing plant somatic cell totipotency. Then, the charge and subsequent discharge rhythm of the SELTP‐associated battery triggered cell totipotency fate and the acquisition of embryogenic competence. These results suggested the importance of SELTP for assembling amyloplasts in somatic dedifferentiated cells. These SELTP‐associated organelles serve as powerful batteries that drive embryonic activation. The increased amyloplasts associated with SELTP were likely to be limiting factors during SE. Our findings highlight the significance of the *SELTP* gene for competent cells expressing totipotency and show that SELTP and the corresponding amylase could serve as markers for early detection of the embryonic cell progenitor, enabling an early diagnosis of embryogenic potential.

At the single‐cell level, our findings demonstrate and highlight the importance of SELTP‐assembled amyloplasts for single cell polarization and cell totipotency determination during transdifferentiation for the acquisition of embryogenic competence. This provides new information that helps to identify embryogenic cells and direct future strategies for SE induction, holding great promise for its advancement in recalcitrant plant species.

## Conflict of interest

The authors declare no conflict of interest.
